# Human Histology

**Published:** 1858-03

**Authors:** 


ART. V.—*Human Histology in its Relations to Descriptive Anatomy, Physiology, and Pathology.* By E. R. PEASLEE, M.D., Professor of Physiology and Pathology in the New York Medical College, etc. etc. Philadelphia: Blanchard & Lea, 1857, 8vo. pp. 616.

THE aim of this excellent compendium, which, as far as we are aware, is the first complete manual or text-book upon histology which has emanated from an American pen, is to give a connected view of the simple chemical elements, of the immediate principles, the simple structural elements, and the proper tissues, entering into the composition of the fluids and solids of the human body; and to associate with the structural elements and the tissues their function while in health, and the changes they undergo in disease.

The work is divided into two parts. The first comprises the classification and description of the simple, chemical elements, and of the primary organic compounds, or immediate principles of which the various tissues and fluids of the human body are composed. The second part is devoted to the description of the tissues and fluids.

Dr. Peaslee arranges all the immediate principles into two groups. The first includes those principles which are crystallizable or volatile without decomposition, and is divided into two classes, viz.:—1. Principies of mineral origin, embracing various gases and salts; and 2. Principles of organic origin formed within the body by dis-assimilation or metamorphosis, and comprising certain acid and saline matters, such as lactic, uric, and hippuric acids, oxalate of lime, lactate of soda, etc., and all the neutral nitrogenized and non-nitrogenized, as well as the fatty and saponaceous compounds. In the second great group are classed all those principles which are not crystallizable or not volatile, except in consequence of decomposition. This group contains fewer substances than the first. It is made up of certain organic, coagulable principles, some of which are naturally liquid, as fibrin, albumen, albumenose, causein, pancreatin, mucosin, and ptyalin; others, solid or semi-solid, as globulin, crystallin, musculin, elasticin, cartilagein, ostein, and keratin; and others again are pigmentary, as hæmatin, biliverdin, melanin, and urrosacin.

The many substances enumerated in these two groups are each briefly and accurately described, and in a manner, moreover, that cannot fail to make the student well acquainted with the elementary composition of the body. The origin and uses of many of these substances, and the known pathological changes which they undergo in the body during the constant disintegration and reconstruction of the tissues, are also concisely stated.

The second portion of the volume is devoted to minute or microscopic anatomy. In the language of our author, "It is a treatise on Human Histology, both general and special, and, at the same time, both physiological and pathological." The first division of this part is occupied with descriptions of the simple histological elements, their distribution, development, etc. The second treats of the fluids of the human body which contain histological elements; and the third, of the tissues properly so-called.

The various structural forms presented to the histologist in the solids and fluids of the human fabric are discussed by Dr. Peaslee under the following heads:—1st. Homogeneous substance; 2d. Simple membrane; 3d. Simple fibre; 4th. Cells; 5th. The tissues proper.

*Homogeneous substance*, called also homogeneous matrix and hyaline substance, is a peculiar, structureless, and more or less solid matter, reresembling coagulated albumen, and found abundantly distributed in various parts of the body. It occupies the spaces between the cells of compound tissues, as cartilage and fibro-cartilage; constitutes a large part of cancerous masses; forms one of the elements of tubercle, and abounds in bone. It originates probably from the albumen of the blood, and serves to connect together various histological elements.

Simple, or *limitary* membrane as it is sometimes called, is a thin, delicate, and structureless layer of what appears to be solidified albumen. In it the microscope can detect neither pores nor openings of any kind whatever. Like the homogeneous, anatomical element, its optical properties are of a negative character. Its vital properties, however, are important, and its distribution extensive. It forms the posterior layer of the capsule of the crystalline lens, the walls of all cells, the myolemma of muscular, and the neurilemma of nervous fibres and tubes, and as a constituent of epithelium and primary, or basement membrane, it lines blood-vessels and lymphatics, and enters the lobes and follicles of all glands.

Dr. Peaslee, somewhat unnecessarily, as it appears to us, restricts the term *simple fibre* to those fibrous threads whose interlacement constitutes the *membrana putaminis*, or lining membrane of the egg-shell, or which are formed in coagulating fibrine as the result of the process known as fibrillation. Such a use of the term is objectionable, because it is thus rendered inapplicable to the white fibrous and yellow elastic tissues, which have always been selected as the best representatives of the simple fibre. This anatomical element, according to our author, "Is not a prominent constituent of the human body, but must be regarded as a merely temporary element, laid down as a framework on which higher histological elements may be developed, and which then becomes absorbed and disappears."

Chapter IV. is taken up with a description of cells, and their component structural elements. The endogenous, exogenous, and fissiparous methods of cell development are successively passed in review. Pathological developments of cell-nuclei, the growth and functions of cells, and the nature of their contents, and a descriptive account of pigment and cancer cells, complete this division of the book. The following varieties of the cancer-cell are recognized and described :—

1. The polygonal, or more or less spherical and ovoid cell.
2. The caudated cell.
3. The fusiform cell.
4. The concentric cell.
5. The compound, or mother cell.
6. Agglomerated nuclei, connected by granular homogenous substance.

In regard to the value of the microscope in the diagnosis of cancer, our author advocates in substance the judicious opinions originally advanced by Lebert, and now generally adopted.

The second division treats of the fluids of the human body. It is divided into four chapters, which are appropriated to the following subjects respectively :—1. The histological relations of the blood, including lymph and chyle. 2. Serous secretions, transudations, and exudations. 3. Mucous and glandular secretions. 4. Cutaneous secretions.

The *tissues* constitute the subject-matter of the third and last division, which occupies more than half the volume. Our author divides the tissues into the *simple,* composed of one histological element, whether cells, fibres, or membranes ; and the *compound,* consisting of two or more such elements. The compound tissues he subdivides into binary and ternary. Among the simple tissues, epithelium and white and yellow fibrous tissues find a place. Contrary to the views of most microscopists, the bones and teeth are also arranged in this class. The compound tissues include areolar and adipose tissues, cartilage, muscle, nerve, membranes, vessels, the alimentary canal and its apendages, the urinary, sexual, and respiratory apparatus, the ductless glands, and the organs of the senses.

In the three-fold division, general arrangement, and composition of the work under consideration, Dr. Peaslee has taken for his guides the original and justly celebrated works of Robin and Verdeil on the "Immediate Principles, Normal and Morbid, which Constitute the Body of Man and the Mammalia;" of Lehmann on "Physiological Chemistry," and of Kölliker on "Human Microscopic Anatomy." Nor has he been unmindful of the special and more recent labors of Beale, Todd and Bowman, Bennett, Gray and others. It will be seen, therefore, that the volume thus briefly noticed fairly and adequately represents the science of human histology in its present advanced state. It is well "gotten up," and is embellished with numerous wood-cuts, of which none, however, can lay claim to novelty, while many are decidedly old and familiar acquaintances, whose ubiquity and tenacity of life are truly extraordinary.

